# Supplementary material for: The Dipeptide Gly-Pro (GP), Derived from Hibiscus sabdariffa, Exhibits Potent Antifibrotic Effects by Regulating the TGF-β1-ATF4-Serine/Glycine Biosynthesis Pathway
Source: Int J Mol Sci. 2023 Sep 3;24(17):13616. doi: 10.3390/ijms241713616 (PMC10487435; doi:10.3390/ijms241713616)
Supplement: Supplementary file 1 [file ijms-24-13616-s001.zip › ijms-2550780-supplementary.pdf]

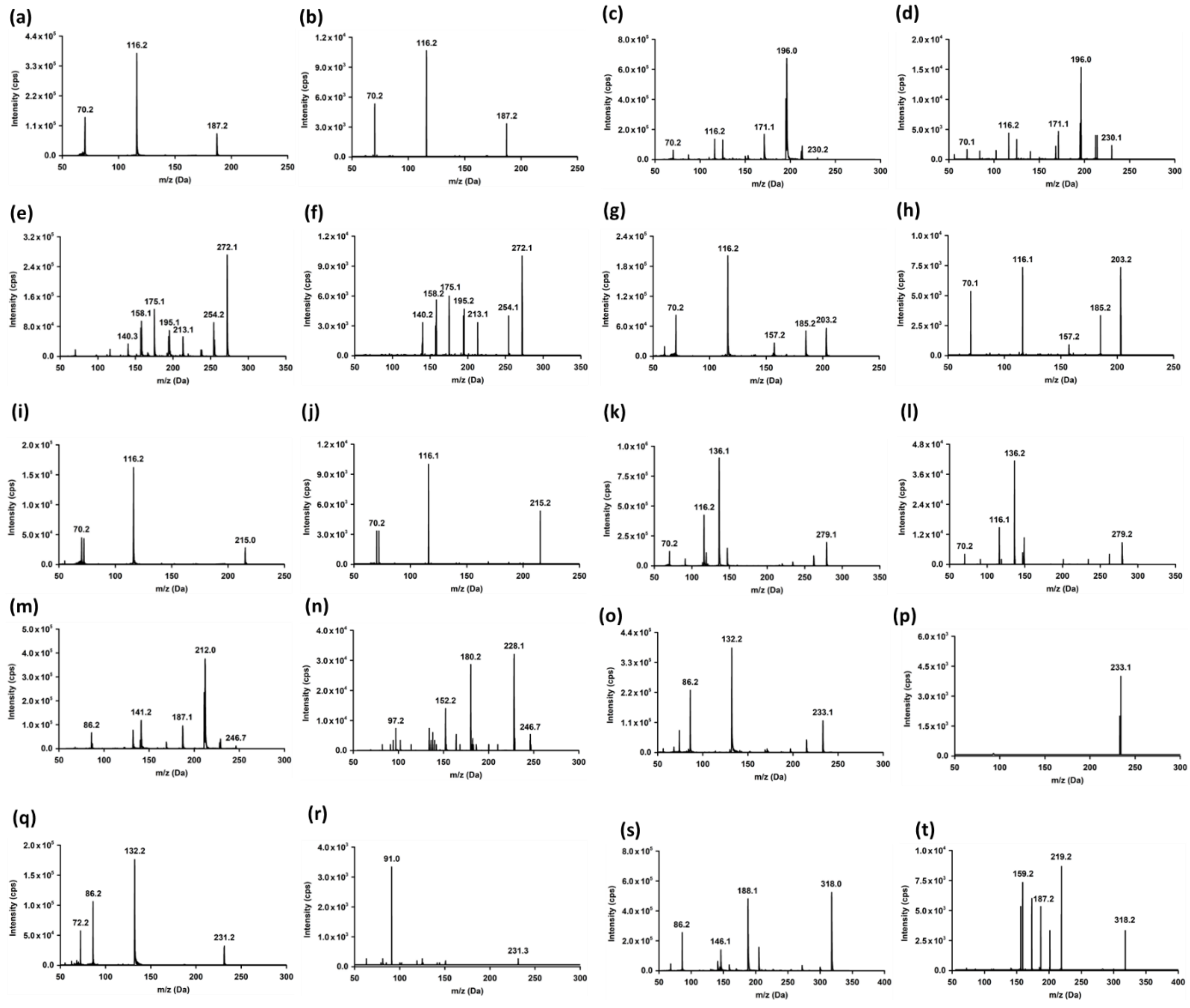

Supplementary Figure S1. Qualitative analysis by comparing the peptides predicted to be present in *Hibiscus sabdariffa* plant cells with the synthesized peptides (standard compounds) through monitoring the MS/MS spectra. (a), (c), (e), (g), (i), (k), (m), (o), (q), (s) represent the MS/MS spectra of the synthesized standard compounds, and (b), (d), (f), (h), (j), (l), (n), (p), (r), (t) correspond to the MS/MS spectra of the peaks expected to be contained in *Hibiscus sabdariffa* plant cells.; (a) AP standard compound (Molecular ion ($[M+H]^+$): 187.2 / Daughter ions: 116.2, 70.2) (b) AP in the extract of *Hibiscus sabdariffa* plant cells (Molecular ion ($[M+H]^+$): 187.2 / Daughter ions: 116.2, 70.2) (c) NP standard compound (Molecular ion ($[M+H]^+$): 230.2 / Daughter ions: 196.0, 171.1, 125.2, 116.2, 70.2) (d) NP in the extract of *Hibiscus sabdariffa* plant cells (Molecular ion ($[M+H]^+$): 230.1 / Daughter ions: 196.0, 171.1, 125.2, 116.2, 70.1) (e) RP standard compound (Molecular ion ($[M+H]^+$): 272.1 / Daughter ions: 254.2, 213.1, 195.1, 175.1, 158.1, 140.3) (f) RP in the extract of *Hibiscus sabdariffa* plant cells (Molecular ion ($[M+H]^+$): 272.1 / Daughter ions: 254.1, 213.1, 195.2, 175.1, 158.2, 140.2) (g) SP standard compound (Molecular ion ($[M+H]^+$): 203.2 / Daughter ions: 185.2, 157.2, 116.2, 70.2) (h) SP in the extract of *Hibiscus sabdariffa* plant cells (Molecular ion ($[M+H]^+$): 203.2 / Daughter ions: 185.2, 157.2, 116.1, 70.1) (i) VP standard compound (Molecular ion ($[M+H]^+$): 215.0 / Daughter ions: 116.2, 72.2, 70.2) (j) VP in the extract of *Hibiscus sabdariffa* plant cells (Molecular ion ($[M+H]^+$): 215.2 / Daughter ions: 116.1, 72.2, 70.2) (k) YP standard compound (Molecular ion ($[M+H]^+$): 279.1 / Daughter ions: 262.0, 147.2, 136.1, 116.2, 70.2) (l) YP in the extract of *Hibiscus sabdariffa* plant cells (Molecular ion ($[M+H]^+$): 279.2 / Daughter ions: 262.1, 147.2, 136.2, 116.1,

70.2) (m) NO standard compound (Molecular ion ([M+H] $^{+}$): 246.7 / Daughter ions: 212.0, 187.1, 141.2, 86.2) (n) NO was not detected in the extract of *Hibiscus sabdariffa* plant cells (Precursor ion ([M+H] $^{+}$): 246.7 / Daughter ions: 228.1, 180.2, 152.2, 97.2) - MS/MS pattern mismatched (o) TO standard compound (Molecular ion ([M+H] $^{+}$): 233.1 / Daughter ions: 132.2, 86.2, 72.2) (p) TO was not detected in the extract of *Hibiscus sabdariffa* plant cells (Precursor ion ([M+H] $^{+}$): 233.1 / Daughter ions: none) - MS/MS pattern mismatched (q) VO standard compound (Molecular ion ([M+H] $^{+}$): 231.2 / Daughter ions: 132.2, 86.2, 72.2) (r) VO was not detected in the extract of *Hibiscus sabdariffa* plant cells (Precursor ion ([M+H] $^{+}$): 231.3 / Daughter ions: 91.0) - MS/MS pattern mismatched (s) OW standard compound (Molecular ion ([M+H] $^{+}$): 318.0 / Daughter ions: 205.1, 188.1, 146.1, 86.2) (t) OW was not detected in the extract of *Hibiscus sabdariffa* plant cells (Precursor ion ([M+H] $^{+}$): 318.2 / Daughter ions: 219.2, 201.3, 187.2, 173.2, 159.2) - MS/MS pattern mismatched

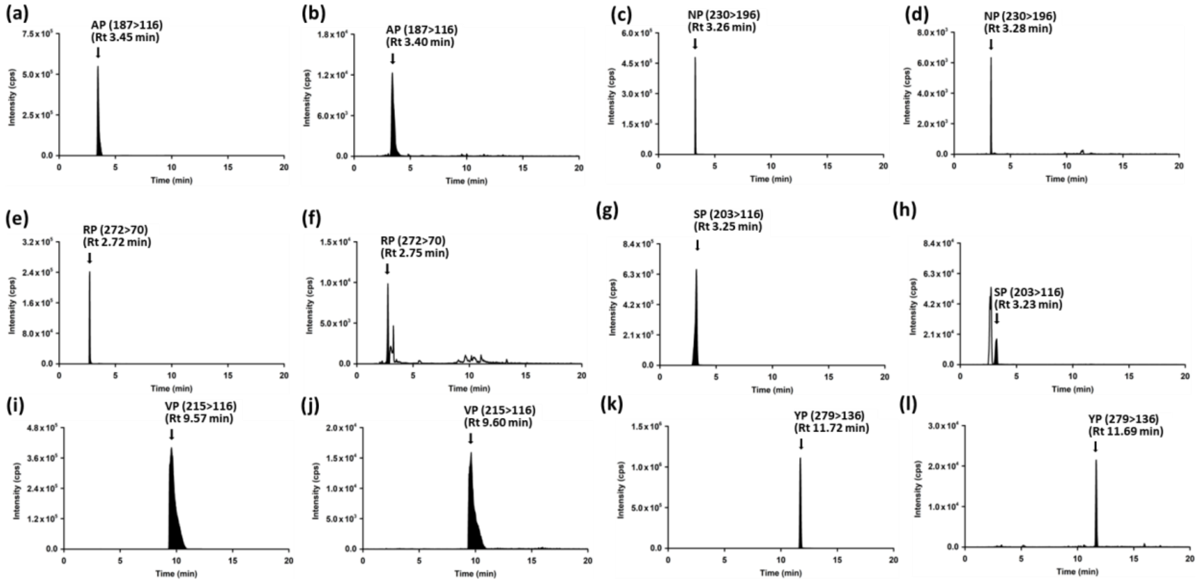

Supplementary Figure S2. Quantitative analysis of 7 peptides through MRM mode.; Using the synthesized peptides as standard materials, the contents of 7 peptides contained in the extract of *Hibiscus sabdariffa* plant cells were measured, respectively, and the corresponding chromatograms were presented. (**a**) AP standard compound (**b**) AP in the extract of *Hibiscus sabdariffa* plant cells (**c**) NP standard compound (**d**) NP in the extract of *Hibiscus sabdariffa* plant cells (**e**) RP standard compound (**f**) RP in the extract of *Hibiscus sabdariffa* plant cells (**g**) SP standard compound (**h**) SP in the extract of *Hibiscus sabdariffa* plant cells (**i**) VP standard compound (**j**) VP in the extract of *Hibiscus sabdariffa* plant cells (**k**) YP standard compound (**l**) YP in the extract of *Hibiscus sabdariffa* plant cells

HPLC conditions	
**Instrument**	1200 Series HPLC system (Agilent, USA)
**Column**	Atlantis dC18 (2.1 × 150 mm, 3 μm, Waters, Ireland)
**Eluent**	Mobile phase A: Water containing 0.1%(v/v) formic acid<br>Mobile phase B: Acetonitrile containing 0.1 %(v/v) formic acid
	Isocratic (Mobile phase A : Mobile phase B = 30 : 70)
**Flow rate**	0.3 mL/min
**Injection volume**	10 μL
**MS conditions**	
**Instrument**	AB SCIEX 3200 QTRAP MS/MS (Applied Biosystems, USA)
**Ionization**	Electrospray Ionization (positive mode)
**Turbo heater temperature (TEM)**	600 °C
**Ion spray voltage (IS)**	5,500 V
**Curtain gas**	10 psi
**Nebulizing gas (GS1)**	55 psi
**Heated gas (GS2)**	45 psi
**Declustering Potential**	22 V (in EPI mode)<br>20 V (in MRM mode)
**Collision Energy**	20 V (in EPI mode)<br>15 V (in MRM mode)

Target ions ($[M+H]^+$) for peptide qualitative analysis in EPI mode											
**Peptide**	AP	GP	NP	RP	SP	VP	YP	NO	TO	VO	OW
**Targetion**	187	173	230	272	203	215	279	246	233	231	318

Precursor and Daughter ions for peptide quantitative analysis in MRM mode							
**Peptide**	AP	GP	NP	RP	SP	VP	YP
**Precursor ion > Daughter ion**	187 > 116	173 > 116	230 > 196	272 > 70	203 > 116	215 > 116	279 > 136

Supplementary Table S1. The conditions for HPLC-MS/MS analysis.

Qualitative and quantitative analysis of dipeptides present in hibiscus were carried out using HPLC-MS. For the qualitative analysis of peptides, it was monitored in the enhanced product ion mode, and the quantitative analysis was performed through multi reaction monitoring mode. The instrumental parameters for analysis are as described above.
